# *Toxoplasma gondii* Antibodies in Raw Milk and Sera of Cows in China

**DOI:** 10.3390/pathogens11101079

**Published:** 2022-09-22

**Authors:** Yu-Min Liu, Yang-Yang Zhang, Lu Wang, Hai-Yang Wang, Chun-Hua Li, Yu-Hang Jiang, Wu-Wen Sun

**Affiliations:** 1 College of Animal Science and Technology, Jilin Agricultural Science and Technology University, Jilin 132101, China; 2 College of Animal Science and Technology, Jilin Agricultural University, Changchun 130118, China; 3 Intelligent Research Center of Cancer Hospital Affiliated to Chongqing University, Chongqing 400030, China; 4 College of Veterinary Medicine, Northwest A&F University, Yangling 712100, China

**Keywords:** cow milk, *Toxoplasma gondii*, seroprevalence, China

## Abstract

*Toxoplasma gondii* is a worldwide food-borne protozoa that has harmful influences on animal and human health. Raw milk containing *T. gondii* has been considered as one of the possible infectious sources for humans. Although China is one of the world’s leading milk consumers, there is still no study to investigate the seroprevalence of *T. gondii* in raw cow milk in China; especially for cows in rural areas. Thus, we conducted this study to examine the specific anti-*T. gondii* IgG-antibody in the raw milk and sera of domestic cows in China. In total, 894 cows were randomly selected from rural areas in northeastern China. The positive rate of *T. gondii* in the milk and serum samples were 6.38% (57/894) and 7.16% (64/894), respectively. Moreover, a history of abortion (OR = 2.03, 95% CI: 1.11–3.72, *p* = 0.022) was identified as the only risk factor for *T. gondii* infection in the studied cows. This study investigated the seroprevalence of *T. gondii* in the raw milk and sera of cows in China; it provided timely and useful data for public health and food safety, especially in rural areas.

## 1. Introduction

*Toxoplasma gondii* is an important food-related parasitic causative agent; it is believed to infect almost the whole species of warm-blooded animals, including humans, terrestrial and aquatic mammals [1,2,3]. Feline and other warm-blooded animals are the definitive and intermediate hosts for this parasite, respectively [1]. The most relevant impact of this parasitic infection is mainly on immune-compromised individuals or pregnant women [1]. This parasitic infection gives rise to huge public health safety problems and economic losses in the veterinary field as well [1].

Until now, *T. gondii* DNA and anti-*T. gondii* antibodies have already been detected in the milk of some kinds of animals, such as goat [4,5], sheep [6], donkey [7], cow [8], buffalo [9], camel [10], cat [11], and even human [4]. Thus, it is believed that the transmission of *T. gondii* can occur via the milk of all animals if consumed raw [12]. Indeed, humans have been reported to present with symptoms of acute *T. gondii* infection after drinking raw goat milk [13]. However, it is believed that it is extremely unlikely to be infected by *T. gondii* via consuming cow milk because cow has already been considered as a poor host for this parasite. Surprisingly, seroepidemiology surveys suggested that the consumption of raw cow milk was significantly related to *T. gondii* infection [14,15].

In China, the demand for milk is ever-increasing and forecast analysis suggests that the demand will be an increase of over 3× compared with 2010 [16]. Thus, it is very important that public health understands the contamination situation of this parasite in raw cow milk. Unfortunately, at present, relevant information is still unavailable. Therefore, we firstly conducted this study to investigate the prevalence of an anti-*T. gondii* IgG-antibody in raw cow milk collected from rural areas in China; this is because rural areas are considered to be important risk areas. The present data will provide new evidences about cow milk contaminated by *T. gondii* in China; in addition, the date highlight that more monitoring and prevention measures should be taken to safeguard the safety of dairy products.

## 2. Results

In this study, 6.38% (57/894) of milk samples and 7.16% (64/894) of serum samples were identified to be positive for *T. gondii*, respectively. Of these, seven negative milk samples tested positive in their correspondent serum samples. A high correlation (Spearman’s coefficient = 0.804, *p*-value < 0.001; and Kendall’s tau = 0.621, *p*-value < 0.001) between the S/P% values acquired in the serum and milk samples was found. Moreover, significant agreements between serum and milk were found among all the studied groups (Table 1).

**Figure 1:**
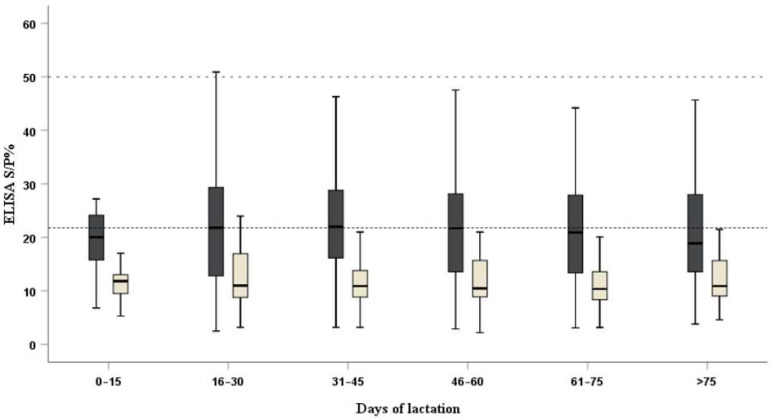
ELISA S/P% values of the cow milk (yellow) and serum (black) samples among the different days of lactation; 21.8 (dotted line) and 50 (dashed line) were the cut-off values for the positive milk and serum samples, respectively.

In addition, a similar trend in antibody levels in milk and serum was found in the present study: in the first half month, the ELISA S/P% values of the milk and serum samples were both low; subsequently, in the second half month (16–30 DL), the S/P% values increased to a peak both in the milk and serum samples. The decline time of seric IgG was the thirtieth day of lactation. From 31 to 75 days after the beginning of lactation, the level of seric IgG fluctuated slightly in both the sera and milk samples (Figure 1).

In this study, the examined cows were divided into five age groups. The highest *T. gondii* prevalence in the studied milk and serum samples were both found in the age group (>60 Mon) without a significant difference (Table 2). During the univariate analysis, a history of abortion was found to be related to *T. gondii* seroprevalence in both the milk (*p* = 0.030) and serum (*p* = 0.045) samples (Table 2). After the multivariate regression analysis, a history of abortion was also the only variable that was inferred to be related to *T. gondii* prevalence in the investigated cows (OR = 2.03, 95% CI: 1.11–3.72, *p* = 0.022) (Table 3).

## 3. Discussion

Due to the abundant production and low production cost, cow milk is the most consumed milk all over the world; accordingly, it can provide essential substances for organism development, including minerals, proteins, and vitamins [9,16]. It is widely believed that it is unlikely to become infected with *T. gondii* through consuming cow’s milk because cows are recognized as a poor host for *T. gondii*. In recent years, a study conducted in Brazil found that the ingestion of untreated cow’s or/and goat’s milk is popular in seropositive pregnant women [17]. Constantly, the ingestion of raw cow’s milk is found to be highly related with *T. gondii* infection [15,18,19]. Thus, the investigation of the prevalence of *T. gondii* in cow’s milk is very important; especially for domestic cows, which is of great significance to food safety. Consequently, we conducted the present study for the first time to investigate the prevalence of *T. gondii* in cow’s milk in China; this will give us valuable data for formulating relevant prevention and control measures for toxoplasmosis in cows and humans.

In this study, anti-*T. gondii* IgG-antibodies were identified in 6.38% (57/894) cow milk samples; this was lower than the 15.9% (OR = 2.77, 95% CI: 1.34–5.73, *p* < 0.05) detected in cows by PCR in Poland [20]; the 11.38% (OR = 1.89, 95% CI: 1.02–3.50, *p* < 0.05) in Iran by ELISA [21]; and the 10.7% (OR = 1.77, 95% CI: 0.99–3.17, *p* > 0.05) in Iranian dairy farms by ELISA [22]. However, it was higher than the 4.00% (OR = 0.61, 95% CI: 0.29–1.30, *p* > 0.05), 4.00% (OR = 0.61, 95% CI: 0.29–1.30, *p* > 0.05), 3.00% (OR = 0.45, 95% CI: 0.19–1.07, *p* > 0.05), and 3.50% (OR = 0.53, 95% CI: 0.24–1.19, *p* > 0.05) in the raw milk of bovines in Iran by cell cultivation, cat bioassay, capture ELISA, and PCR, respectively [9]. Moreover, anti-*T. gondii* IgG-antibodies were detected in 7.16% (64/894) of the serum samples; this is lower than the estimated 10.1% (OR = 1.56, 95% CI: 1.21–2.02, *p* < 0.05) pooled *T. gondii* seroprevalence in cattle in China [23]. Many elements may contribute to these differences, such as different diagnostic methods, sample size, animal welfare, living conditions, cat populations in the neighborhood, and food source [23].

It is well known that severe reproductive problems in small ruminants could be induced by *T. gondii* infection; which can cause huge economic losses in animal husbandry [24,25]. More worrisome, the contamination of *T. gondii* was found on animal by-products, including meat, milk, and other processed products; this is a major hidden issue for public health [26]. Moreover, the contamination will greatly influence global livestock production [27]. Several studies have showed the relation between *T. gondii* infection and the rate of abortion in cows [28,29,30]. Dubey et al. indicated that abortion in cows would only be associated with a primary infection [28]. One cause of abortion and congenital toxoplasmosis in cattle was considered to be maternal toxoplasmosis [29]. Similarity, a history of abortion was confirmed to be highly related with the seroprevalence of *T. gondii* in both the serum and milk samples in the present study. It is noteworthy that another very important protozoan, *Neospora caninum*, cannot be disregarded, it has been considered to play a crucial role in the reproductive failure of cattle and widely distributed in China [31]. Thus, it is necessary to prepare and implement routine surveillance of *T. gondii* and *N. caninum* infection at all levels, including individual, herd, and farm levels. Moreover, farms with history of miscarriages should be more concerned.

Children living in rural areas are at a high risk of *T. gondii* infection because the ingestion of unpasteurized milk is frequent; this is recognized as a possible pathway for *T. gondii* transmission [32]. Clinical evidence shows that a breast-fed child became infected from a *T. gondii*-positive mother, suggesting the important role of milk in the transmission of *T. gondii* [33]. Tachyzoites in the milk and suckling trauma were recognized to make for the transmission of *T. gondii* [33]. Moreover, *T. gondii* cysts could be harbored in relatively stable mammary cells during prelactation. The mammary gland cells could also secrete these silent cysts via exocytosis [34], resulting in *T. gondii* infection via the ingestion of milk. Thus, rural areas may be more affected by *T. gondii* because the pasteurization of milk is unlikely to be implemented due to objective conditions [35].

We are not aware of any simpler studies in China and the present study reports a seroprevalence of 6.38% (57/894) of *T. gondii* in cow milk in China. A history of abortion was confirmed to be significantly related to the *T. gondii* infection in the studied cows. However, one of the most important limitations should not be neglected. We did not calculate the number of necessary samples through a statistical point of view. Thus, the present results could not be used as representative of all the dairy cows in the study area. Moreover, we only detected the IgG antibodies of the samples and it is still not certain that live strains exist in the samples. However, the data of this study is valuable for public health and food safety. Thus, future studies should be conducted to explain the unresolved issues; in addition, it is necessary to prepare and implement relevant prevention and control measures, especially in rural areas.

## 4. Materials and Methods

### 4.1. Ethical Certificate

The study was approved by the Laboratory Animal Welfare and Ethics Committee of Jilin Agricultural Science and Technology University. The owners of domestic cows gave us the permission to collect the samples. In this case, the owners of the domestic cows were invited to collect the milk samples; and the serum samples were collected by local veterinarians.

### 4.2. Sampling

**Figure 2:**
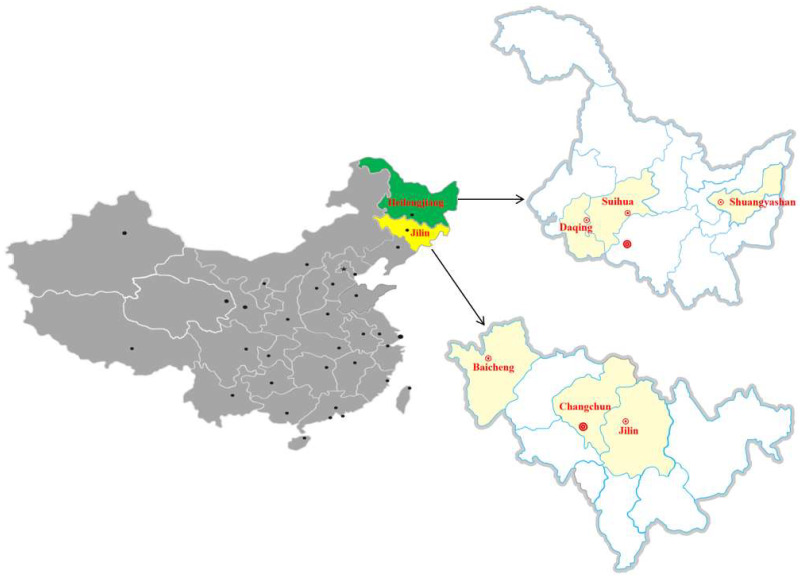
Geographic positions where this study was conducted. Dot represents provincial capital.

The present study was conducted in three cities (Changchun, Jilin, and Baicheng) of the Jilin province (40°50′–46 °19′ N; 121°38′–131°19′ E); and three cities (Daqing, Shuangyashan, and Suihua) of the Heilongjiang province (43°26′–53°33′ N; 121°11′–135°05′ E), in northeastern China between January 2019 and September 2020 (Figure 2). At last, 894 domestic cows were randomly selected as target animals for this study to collect the serum and milk. In this study, all the included cows were kept to produce milk for sale or self-supply. The first step was to collect the milk samples due to its convenience.

Approximately 10 mL of milk was collected from each cow after disinfecting the udder.

Subsequently, blood samples were collected; a blood sample of approximately 5 mL was obtained from the jugular vein of each cow and stored in a blood collecting tube. After sampling, all the collected samples were kept in medical incubator; and immediately delivered to a laboratory. We firstly isolated the serum from the blood samples according to our previous study [5] and stored them at −20 °C. Then, we processed the collected milk samples, and removed the fatty components and the somatic cells based on the reported procedure [36]; we kept the remaining ingredients at −20 °C until they were used. The individual data of each cow was obtained from the owner by oral inquiry. We referred to the method of the previous study to calculate the day of lactation [37].

### 4.3. Serologic Examination

In this study, we used a commercial enzyme-linked immunosorbent assay (ELISA) kit (ID Screen^®^ Toxoplasmosis Indirect MultiSpecies, IDVET, Montpellier, France) to test the specific anti-*T. gondii* IgG-antibodies in the milk and serum samples according to the reported procedure [32]. For the positive criteria, an S/P% of the serum samples of ≥50% (sample to positive ≥50% = positive) and an S/P% of the milk samples of ≥21.8% (sample to positive ≥21.8% = positive) were considered as positive [38].

### 4.4. Statistical Analysis

In this study, a SPSS 25.0 software package was employed to perform the statistical analysis. The cut-off value of statistically significant was a *p*-value < 0.05. Moreover, Spearman and Kendall rank correlation coefficients were used to evaluate the correspondence between all the serum and milk results according to the day of lactation. A chi-squared test was used to evaluate the univariate associations between *T. gondii* seroprevalence in the domestic cows and the risk factors. Finally, we identified the possible risk factors for *T. gondii* infection in the included cows by multivariate logistic regression analysis.

## Figures and Tables

**Table 1:** Variation of the agreement between the ELISA S/P% results obtained from the cow’s milk and serum samples during lactation.

Statistical Analysis	Days Postpartum					
	0–15	16–30	31–45	46–60	61–75	>75
Kendall’s Tau (*p*-value)	0.644 (0.000)	0.687 (0.000)	0.605 (0.000)	0.598 (0.000)	0.593 (0.000)	0.583 (0.000)
Spearman’s coefficient (*p*-value)	0.796 (0.000)	0.867 (0.000)	0.792 (0.000)	0.772 (0.000)	0.780 (0.000)	0.751 (0.000)

**Table 2:** Detection of the *Toxoplasma* IgG antibody in the milk and serum samples according to the characteristics of the cows included in the present study.

Variable	Classification	Number	Serum			Milk		
			Positive	Prevalence	*p*-Value	Positive	Prevalence	*p*-Value
Age (month)	≤24	38	3	7.89	0.898	3	7.89	0.873
	25–36	281	18	6.41		16	5.69	
	37–48	193	12	6.22		12	6.22	
	49–60	263	21	7.98		16	6.08	
	>60	119	10	8.40		10	8.40	
Region	Changchun	188	7	3.72	0.164	7	3.72	0.192
	Jilin	153	10	6.54		9	5.88	
	Baicheng	142	9	6.34		7	4.93	
	Shuangyashan	93	6	6.45		5	5.38	
	Daqing	159	17	10.69		16	10.06	
	Suihua	159	15	9.43		13	8.18	
History of abortion	Yes	144	16	11.11	0.045	15	10.42	0.030
	No	750	48	6.40		42	5.60	
Days postpartum	0–15	21	1	4.76	0.374	1	4.76	0.709
	16–30	228	15	6.58		15	6.58	
	31–45	164	6	3.66		6	3.66	
	46–60	259	24	9.27		19	7.34	
	61–75	148	12	8.11		10	6.76	
	>75	74	6	8.11		6	8.11	
Cat in house	Yes	262	24	9.16	0.135	21	8.02	0.196
	No	632	40	6.33		36	5.70	
Type of drinking water	Well water	276	22	7.97	0.529	21	7.61	0.313
	Tap water	618	42	6.80		36	5.83	
Type of fodder	Forage	581	45	7.75	0.354	39	6.71	0.575
	Forage/commercial feed	313	19	6.07		18	5.75	
Total		894	64	7.16		57	6.38	

**Table 3:** Multivariate logistic regression for the risk factors of *T. gondii* infection.

Variable	Odds Ratio (95% Confidence Internal)	*p*-Value
Age (month) (≤24 vs. >60)	1.37 (0.35–5.32)	0.647
Age (month) (25–36 vs. >60)	0.90 (0.40–2.02)	0.800
Age (month) (37–48 vs. >60)	0.87 (0.36–2.12)	0.761
Age (month) (49–60 vs. >60)	0.98 (0.44–2.17)	0.964
Region (Changchun vs. Suihua)	0.40 (0.16–1.01)	0.053
Region (Jilin vs. Suihua)	0.72 (0.31–1.66)	0.442
Region (Baicheng vs. Suihua)	0.67 (0.29–1.58)	0.360
Region (Shuangyashan vs. Suihua)	0.72 (0.27–1.93)	0.517
Region (Daqing vs. Suihua)	1.16 (0.56–2.41)	0.687
History of abortion	2.03 (1.11–3.72)	0.022
Days postpartum (0–15 vs. >75)	0.58 (0.07–5.13)	0.625
Days postpartum (16–30 vs. >75)	0.76 (0.28–2.03)	0.577
Days postpartum (30–45 vs. >75)	0.41 (0.13–1.32)	0.133
Days postpartum (41–60 vs. >75)	1.17 (0.43–3.00)	0.738
Days postpartum (61–75 vs. >75)	1.06 (0.38–2.93)	0.913
Cat in house	1.42 (0.84–2.42)	0.193
Type of drinking water	1.16 (0.68–1.99)	0.591
Type of fodder	1.30 (0.75–2.26)	0.358

## Data Availability

The data presented in this study are available on request from the corresponding author.

## References

[1] Dubey J.P. (2010). Toxoplasmosis of Animals and Humans.

[2] Huang J.F., Wu Y.L., Wang M.F., Zhu Y.Y., Lin S. (2022). Lower vitamin D levels are associated with higher seroprevalence of *Toxoplasma gondii*—A US national survey study. Zoonoses.

[3] Li M.Y., Gao X.N., Ma J.Y., Elsheikha H.M., Cong W. (2022). A systematic review, meta-analysis and meta-regression of the global prevalence of *Toxoplasma gondii* infection in wild marine mammals and associations with epidemiological variables. Transbound. Emerg. Dis..

[4] Khamsian E.M., Hajimohammadi B., Eslami G., Fallahzadeh M.H., Hosseini S.S. (2021). *Toxoplasma gondii* in milk of human and goat from the Desert Area in central Iran. Iran. J. Parasitol..

[5] Liu Y.M., Wang L., Wang H.Y., Li C.H., Jiang Y.H., Sun W.W. (2021). First detection of anti-*Toxoplasma gondii* antibodies in domestic goat’s serum and milk during lactation in China. Microb. Pathog..

[6] Luptakova L., Benova K., Rencko A., Petrovova E. (2015). DNA detection of *Toxoplasma gondii* in sheep milk and blood samples in relation to phase of infection. Vet. Parasitol..

[7] Chen L., Zhao Z.J., Meng Q.F. (2021). Detection of specific IgG-antibodies against *Toxoplasma gondii* in the serum and milk of domestic donkeys during lactation in China: A potential public health concern. Front. Cell. Infect. Microbiol..

[8] Koethe M., Schade C., Fehlhaber K., Ludewig M. (2017). Survival of *Toxoplasma gondii* tachyzoites in simulated gastric fluid and cow’s milk. Vet. Parasitol..

[9] Dehkordi F.S., Borujeni M.R., Rahimi E., Abdizadeh R. (2013). Detection of *Toxoplasma gondii* in raw caprine, ovine, buffalo, bovine, and camel milk using cell cultivation, cat bioassay, capture ELISA, and PCR methods in Iran. Foodborne Pathog. Dis..

[10] Iacobucci E., Taus N.S., Ueti M.W., Sukhbaatar L., Bastsukh Z., Papageorgiou S., Fritz H. (2019). Detection and genotypic characterization of *Toxoplasma gondii* DNA within the milk of Mongolian livestock. Parasitol. Res..

[11] Powell C.C., Brewer M., Lappin M.R. (2001). Detection of *Toxoplasma gondii* in the milk of experimentally infected lactating cats. Vet. Parasitol..

[12] EFSA (2007). Surveillance and monitoring of *Toxoplasma* in humans, food and animals: Scientific opinion of the Panel on Biological Hazards. EFSA J..

[13] Sacks J.J., Roberto R.R., Brooks N.F. (1982). Toxoplasmosis infection associated with raw goat’s milk. JAMA.

[14] Alvarado-Esquivel C., Liesenfeld O., Torres-Castorena A., Estrada-Martinez S., Urbina-Alvarez J.D., Ramos-de la Rocha M., Marquez-Conde J.A., Dubey J.P. (2010). Seroepidemiology of *Toxoplasma gondii* infection in patients with vision and hearing impairments, cancer, HIV, or undergoing hemodialysis in Durango, Mexico. J. Parasitol..

[15] da Silva M.G., Vinaud M.C., de Castro A.M. (2014). Epidemiological factors associated with seropositivity for toxoplasmosis in pregnant women from Gurupi, State of Tocantins, Brazil. Rev. Soc. Bras. Med. Trop..

[16] Bai Z., Lee M.R.F., Ma L., Ledgard S., Oenema O., Velthof G.L., Ma W., Guo M., Zhao Z., Wei S. (2018). Global environmental costs of China’s thirst for milk. Glob. Chang. Biol..

[17] Moura F.L., Amendoeira M.R., Bastos O.M., Mattos D.P., Fonseca A.B., Nicolau J.L., Neves L.B., Millar P.R. (2013). Prevalence and risk factors for *Toxoplasma gondii* infection among pregnant and postpartum women attended at public healthcare facilities in the city of Niteroi, state of Rio de Janeiro, Brazil. Rev. Soc. Bras. Med. Trop..

[18] Elsheikha H.M., Azab M.S., Abousamra N.K., Rahbar M.H., Elghannam D.M., Raafat D. (2009). Seroprevalence of and risk factors for *Toxoplasma gondii* antibodies among asymptomatic blood donors in Egypt. Parasitol. Res..

[19] Heukelbach J., Meyer-Cirkel V., Moura R.C., Gomide M., Queiroz J.A., Saweljew P., Liesenfeld O. (2007). Waterborne toxoplasmosis, northeastern Brazil. Emerg. Infect. Dis..

[20] Cisak E., Zając V., Sroka J., Sawczyn A., Kloc A., Dutkiewicz J., Wójcik-Fatla A. (2017). Presence of pathogenic Rickettsiae and protozoan in samples of raw milk from cows, goats, and sheep. Foodborne Pathog. Dis..

[21] Razmi G., Barati M. (2017). Prevalence of *Neospora caninum* and *Toxoplasma gondii* antibodies in bulk milk of dairy cattle, Mashhad, Iran. Arch. Razi Inst..

[22] Gharekhani J., Yakhchali M., Afshari A., Adabi M. (2021). Herd-level contamination of *Neospora caninum*, *Toxoplasma gondii* and *Brucella* in milk of Iranian dairy farms. Food Microbiol..

[23] Gong Q.L., Li J., Li D., Tian T., Leng X., Li J.M., Shi K., Zhang N.Z., Du R., Zhao Q. (2020). Seroprevalence of *Toxoplasma gondii* in cattle in China from 2010 to 2019: A systematic review and meta-analysis. Acta Trop..

[24] Nayeri T., Sarvi S., Moosazadeh M., Daryani A. (2021). Global prevalence of *Toxoplasma gondii* infection in the aborted fetuses and ruminants that had an abortion: A systematic review and meta-analysis. Vet. Parasitol..

[25] Sah R.P., Dey A.R., Rahman A.K.M.A., Alam M.Z., Talukder M.H. (2019). Molecular detection of *Toxoplasma gondii* from aborted fetuses of sheep, goats and cattle in Bangladesh. Vet. Parasitol. Reg. Stud. Rep..

[26] Almeria S., Dubey J.P. (2021). Foodborne transmission of *Toxoplasma gondii* infection in the last decade. An overview. Res. Vet. Sci..

[27] Stelzer S., Basso W., Silv’an J.B., Ortega-Mora L., Maksimov P., Gethmann J., Conrathsa F.J., Schares G. (2019). *Toxoplasma gondii* infection and toxoplasmosis in farm animals: Risk factors and economic impact. Food Waterborne Parasitol..

[28] Dubey J.P. (1998). Advances in the life cycle of Toxoplasma gondii. Int. J. Parasitol..

[29] Wiengcharoen J., Thompson R.C., Nakthong C., Rattanakorn P., Sukthana Y. (2011). Transplacental transmission in cattle: Is Toxoplasma gondii less potent than Neospora caninum?. Parasitol. Res..

[30] Esteban-Redondo I., Innes E.A. (1997). Toxoplasma gondii infection in sheep and cattle. Comp. Immunol. Microbiol. Infect. Dis..

[31] Wei X.Y., An Q., Xue N.Y., Chen Y., Chen Y.Y., Zhang Y., Zhao Q., Wang C.R. (2022). Seroprevalence and risk factors of *Neospora caninum* infection in cattle in China from 2011 to 2020: A systematic review and meta-analysis. Prev. Vet. Med..

[32] Radon K., Windstetter D., Eckart J., Dressel H., Leitritz L., Reichert J., Schmid M., Praml G., Schosser M., von Mutius E. (2004). Farming exposure in childhood, exposure to markers of infections and the development of atopy in rural subjects. Clin. Exp. Allergy.

[33] Ishag M.Y., Magzoub E., Majid M. (2006). Detection of *Toxoplasma gondii* tachyzoites in the milk of experimentally infected lacting She-Camels. J. Anim. Vet. Adv..

[34] Hiramoto R.M., Mayrbaurl-Borges M., Galisteo A.J., Meireles L.R., Macre M.S., Andrade H.F. (2001). Infectivity of cysts of the ME-49 *Toxoplasma gondii* strain in bovine milk and homemade cheese. Rev. Saude Publica.

[35] Dubey J.P., Murata F.H.A., Cerqueira-Cézar C.K., Kwok O.C.H., Yang Y.R. (2020). Public Health Significance of Toxoplasma gondii Infections in Cattle: 2009–2020. J. Parasitol..

[36] Petruzzelli A., Amagliani G., Micci E., Foglini M., Di Renzo E., Brandi G., Tonucci F. (2013). Prevalence assessment of *Coxiella burnetii* and verocytotoxin-producing *Escherichia coli* in bovine raw milk through molecular identification. Food Control.

[37] Gazzonis A.L., Zanzani S.A., Villa L., Manfredi M.T. (2019). *Toxoplasma gondii* in naturally infected goats: Monitoring of specific IgG levels in serum and milk during lactation and parasitic DNA detection in milk. Prev. Vet. Med..

[38] Gazzonis A.L., Zanzani S.A., Stradiotto K., Olivieri E., Villa L., Manfredi M.T. (2018). *Toxoplasma gondii* antibodies in bulk tank milk samples of caprine dairy herds. J. Parasitol..

